# Diagnosis and Treatment of Leprosy Reactions in Integrated Services - The Patients' Perspective in Nepal

**DOI:** 10.1371/journal.pntd.0002089

**Published:** 2013-03-07

**Authors:** Sonia F. Raffe, Min Thapa, Saraswoti Khadge, Krishna Tamang, Deanna Hagge, Diana N. J. Lockwood

**Affiliations:** 1 London School of Hygiene and Tropical Medicine, London, United Kingdom; 2 Anandaban Leprosy Hospital, Kathmandu, Nepal; 3 Lalgadh Hospital, Dhanush District, Janakpur, Nepal; University of California San Diego School of Medicine, United States of America

## Abstract

Leprosy care has been integrated with peripheral health services, away from vertical programmes. This includes the diagnosis and management of leprosy reactions, which cause significant morbidity. We surveyed patients with leprosy reactions at two leprosy hospitals in Nepal to assess their experience of leprosy reaction management following integration to identify any gaps in service delivery.

**Methods:**

Direct and referral patients with leprosy reactions were interviewed in two of Nepal's leprosy hospitals. We also collected quantitative and qualitative data from clinical examination and case-note review to document the patient pathway.

**Results:**

Seventy-five patients were interviewed. On development of reaction symptoms 39% presented directly to specialist services, 23% to a private doctor, 17% to a district hospital, 10% to a traditional healer, 7% to a health post and 4% elsewhere. Those who presented directly to specialist services were 6.6 times more likely to start appropriate treatment than those presenting elsewhere (95% CI: 3.01 to 14.45). The average delay between symptom onset to commencing corticosteroids was 2.9 months (range 0–24 months). Obstacles to early presentation and treatment included diagnostic challenge, patients' lack of knowledge and the patients' view of health as a low priority. 40% received corticosteroids for longer than 12 weeks and 72% required an inpatient stay. Treatment follow-up was conducted at locations ranging from health posts to specialist hospitals. Inconsistency in the availability of corticosteroids peripherally was identified and 41% of patients treated for leprosy and a reaction on an outpatient basis attended multiple sites for follow-up treatment.

**Conclusion:**

This study demonstrates that specialist services are necessary and continue to provide significant critical support within an integrated health system approach towards the diagnosis and management of leprosy reactions.

## Author Summary

The global strategy for leprosy has moved patient care to general health services with the aim of improving access to treatment. We suspected that leprosy patients with a common complication, leprosy reactions, are not being diagnosed and treated promptly in integrated services. Leprosy reactions cause nerve damage and, if not treated early, can cause significant disability. We interviewed 75 patients with a leprosy reaction in Nepal, a country with a fully-integrated leprosy service. Patients were experiencing average delays of 2.9 months between developing leprosy reactions and starting treatment. Many required extended courses of treatment, an inpatient stay or experienced a recurrence of their reaction. Patients also continue to attend specialist services for both diagnosis and follow-up. Patient care could be improved by utilising specialist knowledge for training and the management of complex cases. Health care workers and patients need to be educated about leprosy reactions. A wider implication of the study is that health policy-makers need to be cautious not to over-simplify medical conditions when restructuring services.

## Introduction

Leprosy reactions play a significant role in the morbidity associated with the disease. These immune-mediated complications, seen in up to 50% of patients [1], can cause rapid nerve damage resulting in anaesthesia and weakness, which in turn increases risk of injury and deformity [2]. Two types of reactions are recognized: type 1 (T1R, also known as reversal or downgrading) and type 2 reactions (erythema nodosum leprosum, ENL). They can occur at presentation, during treatment for leprosy with multi-drug therapy (MDT) and occasionally following completion of MDT [3].

Type 1 reactions, caused by an increase in cell-mediated immunity, result in skin or nerve inflammation at sites of *Mycobacterium leprae* infection. Skin lesions become tender, erythematous and oedematous while nerve involvement produces pain, paraesthesia or a sudden deterioration in function [4], [5]. Prompt and appropriate treatment is essential to prevent permanent neurological deficit with observed recovery rates of 60–70% in those identified and treated within six months of onset [3].

Type 2 reactions (ENL) are immune complex-mediated [1]. Symptoms are diverse with characteristic painful, erythematous subcutaneous nodules occurring with systemic features including fever, lymphadenitis, arthritis, neuritis, iridocyclitis or orchitis [5]. ENL can run a chronic or recurrent course and is an important cause of neuropathy and consequent disability [6].

Both type 1 and ENL reactions require treatment with corticosteroids, in addition to treatment for underlying infection with *Mycobacteria leprae.* While effective standardized treatment exists for leprosy infection (MDT), reaction treatment is challenging. Standardized treatment regimens have been suggested but are supported by little evidence as the correct dose and duration of treatment remains unclear [7], [8]. Treatment is complicated by reaction recurrences commonly seen in both reaction types, and by the chronic course seen in up to 62.5% of ENL patients [6], [9].

In Nepal, leprosy services have been fully integrated into the peripheral setting, away from vertical programmes. This move, as part of the global strategy for leprosy, was made to promote early diagnosis and treatment and to reduce stigma by normalizing leprosy treatment alongside other chronic diseases [10]. The rural population of Nepal could benefit from closer proximity to services. Only 43% live within 2 km of a road and only 6% own a motorized vehicle [11]. Since 1996 leprosy services have been available in health posts and peripheral hospitals throughout the 75 districts of Nepal [12]. Included within the remit of the peripheral health unit is the recognition of leprosy reactions, treatment of mild reactions and the referral of severe reactions to a higher level of care which may be a district hospital or a leprosy specialist service [13].

However, our experience at a referral hospital where patients often appear to have experienced delays in the diagnosis of their leprosy reaction suggests that leprosy reactions are not yet well recognised and managed. This study tests this hypothesis and documents the experience of patients developing a leprosy reaction in Nepal, a country with a formally fully integrated health service. Data collected at interview with patients newly diagnosed with leprosy reaction details the journey that patients in Nepal make through the health service from the development of symptoms, to diagnosis, treatment and follow-up. By documenting this journey we aimed to describe the delays experienced by symptomatic patients, the obstacles preventing prompt initiation of treatment and the experience of those on treatment. We aimed to identify any gaps in current service delivery in order to help strengthen future services to ensure effective management of leprosy reactions.

## Methods

### Ethics Statement

Ethical approval for the study was obtained from the Nepal Health and Research Council and from The London School of Hygiene and Tropical Medicine Ethics Committee. The medical director at each study site also granted permission to access patients. Patients were provided with written and verbal information about the study through a translator experienced in dealing with leprosy patients in the local language (Nepali or Maithali). Participants were able to ask questions and informed that they could withdraw, without consequence, at any point. Witnessed written consent was obtained for all participants.

### Patient Selection

Patients were recruited from two of Nepal's specialist leprosy hospitals and their satellite clinics: Anandaban Hospital, the main referral hospital for the Central region of Nepal, and Lalgadh Hospital, in the Terai (southern flatlands), near the Indian border.

Patients were recruited to the study over two weeks at each site. Patients attending clinics or current inpatients with reaction occurrence within the past five years were approached for enrolment. Patients were eligible to take part in the study if they were 16 years old or over and had been diagnosed with a leprosy reaction by a healthcare worker. To reduce recall bias patients were only included if the onset of their reaction had occurred in the last five years. To reduce selection bias all eligible patients were approached. Because of this study design the sample size was not pre-determined.

### Data Collection

Quantitative and qualitative data were collected at patient interview, through review of case-notes (where available) and by a brief clinical examination. The interview was semi-structured based on a questionnaire tool designed by the authors with support from the staff at Anandaban Hospital. In addition, a free-text chart was also used to collect qualitative data regarding patients' journeys from first symptoms to appropriate treatment. Eighteen months later local staff collected additional data regarding treatment and reaction duration from case notes.

### Data Analysis

Once collected, data were anonymised and stored in an encrypted Microsoft Access database. Epi Info version 3.5.1 was used for analysis. Descriptive statistics were used to present the majority of the results with the Chi-squared test being used to compare the duration of travel of those attending specialist services with those attending elsewhere. Patients were categorised by reaction type (T1R, neuritis or ENL) based on the documented diagnosis in the case-notes. To provide detail on the nature and severity of the reaction episodes they were further categorised into acute single, acute multiple or chronic episodes based on definitions described by Pocaterra et al [9].

The qualitative data were analysed as an ongoing process, beginning at the data collection stage. This method, described in the literature as sequential or interim analysis, allowed for the early identification of themes which were then explored with later participants to identify common experiences or attitudes [14].

## Results

### Patient Details

Seventy-eight reaction patients were identified and 75 took part in the study. Three were excluded; one due to memory problems and two as a translator was not available for their dialect at the time of recruitment.

**Figure 1:**
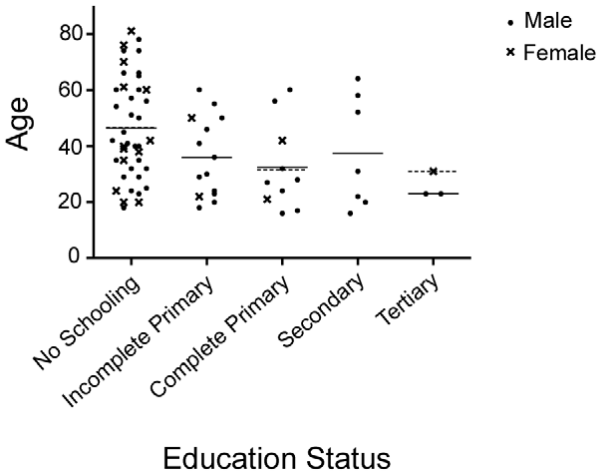
Age, gender and education status of patients.

Fifty-seven (78%) of those interviewed were male. Ages were evenly distributed in range from 16 to 78 years, with a mean age of 40 years. 93% lived in a village or rural location. 55% had never attended school and 49% were farmers (figure 1). Participants came from 27 different districts of Nepal, including 11 districts outside the immediate catchment area served by the two hospitals (data not shown).

Patients had been diagnosed with leprosy between August 2005 and July 2010. Sixty-five (86%) of participants had multibacillary disease and 10 (14%) paucibacillary disease. Forty-nine patients (65%) were first diagnosed with leprosy at a specialist service, 15 at district or general hospitals, four by private doctors, five at health posts and two at other locations.

Of the patients interviewed, 38 (51%) were inpatients receiving reaction treatment while 19 (25%) were attending a specialist service more than once per month, 11 (15%) monthly and four (5%) less than once a month. At the time of interview, three of the patients were newly diagnosed with follow up frequency not yet established.

### Reaction Details

**Figure 2:**
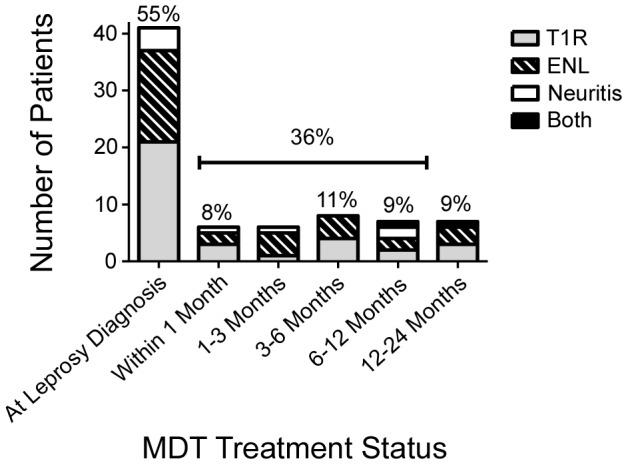
Timing of reaction diagnosis in relation to multi-drug therapy.

Many patients (55%) first presented with reaction and previously undiagnosed leprosy (figure 2). Twenty-four patients were taking MDT at time of reaction diagnosis and six patients had completed their MDT course.

Forty-two patients had a documented T1R (including eight pure neuritis patients), 32 ENL and one patient was documented as having both a T1R and ENL simultaneously (figure 2). Changes in skin lesions were the commonest symptom in T1R and the development of painful nodules was the symptom most frequently reported in ENL, followed by joint or facial pain (table 1). At time of interview, 37% (28) had some evidence of neuropathy by voluntary muscle strength and sensory testing: 35% of T1R (12/34), 35% ENL (11/31) and 62.5% of neuritis patients.

**Table 1:** Symptoms reported by patients with leprosy reactions.

T1R/Neuritis Symptoms	Yes (%)	No (%)	ENL Symptoms	Yes (%)	No (%)
**Lesion change**	30 (70)	13 (30)	Painful skin nodules	28 (85)	5 (15)
**Change in sensation**	20 (47)	23 (53)	Fever	22 (67)	11 (33)
**Pain in elbows/knees/face**	17 (40)	26 (60)	Pain in elbows/knees/face	16 (48)	17 (52)
**Difficulty walking/grasping/facial expressions**	17 (40)	26 (60)	Muscle pain	6 (18)	27 (82)
**New lesions**	14 (33)	29 (67)	Vision changes	4 (12)	29 (88)
**New ulcers**	6 (14)	37 (86)	Eye pain	4 (12)	29 (88)
**Generally weak or unwell**	1 (2)	42 (98)	Generally weak or unwell	9 (27)	24 (73)
**Other**	4 (9)	39 (91)	Testicular pain (males)	2 (6)	31 (94)
			Other	7 (21)	26 (79)

**Figure 3:**
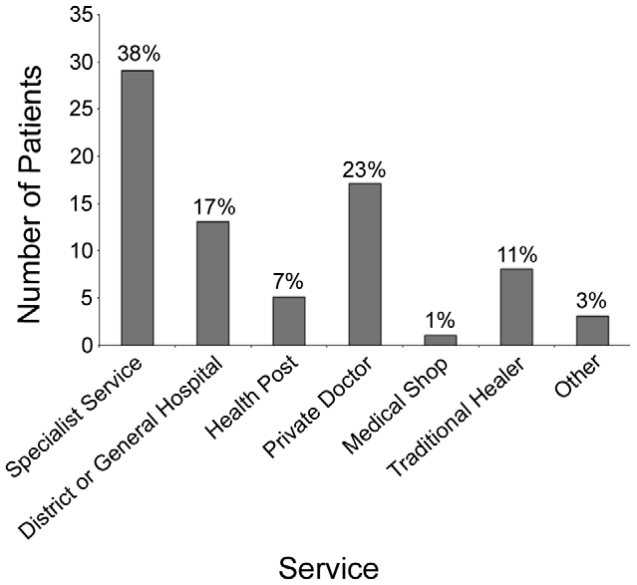
Number of patients initially presenting to each service.

Upon development of leprosy reaction, patients presented to several services. Twenty-nine presented directly to specialist services (including three who were current inpatients), 17 presented to a private doctor, 13 to a district or general hospital, eight to a traditional healer, five to a health post, one to a medical shop and two to other locations (figure 3).

Patients estimated the time they spent travelling in order to consult the above services for initial advice. Most patients first sought medical care for their reaction symptoms somewhere within an hour (51%) or within a day's travel from their home (43%), which is contextually within the range of normal travel in Nepal. Only 4% initially travelled more than a day. Two (3%) were already inpatients for other reasons when their reaction developed. Most patients (56%) travelled by bus when initially seeking advice.

### Experience with Integrated Services

While 29 (39%) of patients interviewed initially sought treatment from leprosy specialty care, 46 (61%) of patients accessed integrated health care services prior to attending leprosy specialty services. This group accessed an average of two facilities (range 1–4) prior to approaching specialty services. Patients did not consistently report how many times they accessed each site, though many mentioned more than once. At some point in their journeys, these patients sought reaction care in one or more of the following health care settings: district or general hospital (23, 54%), private doctor (21, 49%), traditional healer (9, 23%), health post (5, 12%) or other (3, 7%).

Twenty eight (65%) of those initially attending a peripheral service reported at least one misdiagnosis of arthritis, photosensitivity, nerve disease or other skin disease with some receiving treatments including traditional medicine, painkillers, vitamins or methotrexate.

Of the 46 patients initially seeking advice from integrated services 21 (46%) either initially or eventually encountered an integrated health professional who correctly recognized leprosy and/or the reaction and either commenced appropriate treatment of referred the patient to a specialist service.

### Presentation to Specialist Services

Of the 29 patients who presented directly to a specialist service (excluding the three who were inpatients at time of reaction), 17% did so on their own initiative, 34% on advice from another patient with leprosy and 28% on advice from a relative or friend. Eighteen patients in this group had no previous diagnosis of leprosy and were therefore unknown to services. More patients travelled for over one hour to attend specialist services than those seeking advice at a peripheral service (p<0.001).

**Figure 4:**
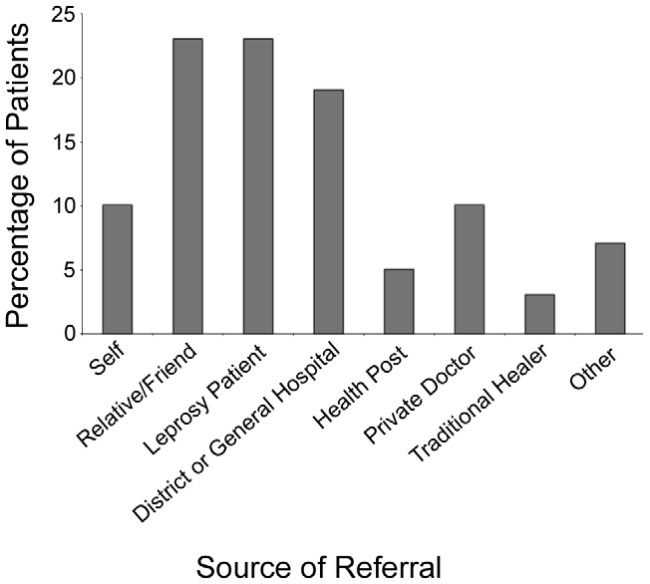
Source of referral to leprosy specialist service.

Due to the nature of the sample all patients eventually received treatment at one of the leprosy specialist services. Over half (56%) attended on their own initiative or following the advice of a friend, relative or former patient. 19% were referred from a district or general hospital, 10% by private doctors 7% from an other or unknown source, 5% from health posts and 3% from traditional healers (figure 4).

### Reaction Treatment

At initial presentation 31 patients were started on steroids. Of the 29 patients who presented directly to a specialist service 25 (86%) received steroids. Of the 18 patients who presented to a government facility (health post or district/general hospital) five (28%) received steroids. One further patient was given steroids from an unknown location. Overall, patients who presented to a specialist service were 6.6 times more likely to be started on steroids than those seeking help elsewhere i.e. RR 6.6 (95% CI: 3.01 to 14.45).

Nine patients seen in the peripheral setting were immediately referred to a specialist service at first presentation. Sixty-five (87%) did not receive steroids until arrival at a specialist service. Once seen at a specialist service, 69 (92%) received steroids that day.

**Figure 5:**
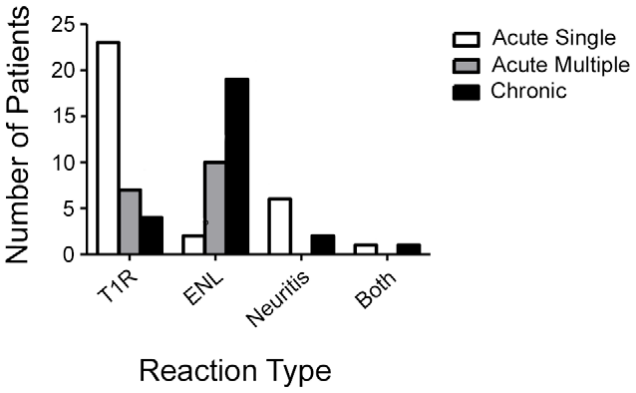
Frequency of reaction by type of episode.

Details regarding treatment duration were available for 67 patients. Nine patients were successfully treated with a 12 week course of steroids and a further 14 patients (34% in total) responded within 20 weeks. Only two ENL patients were successfully managed within 20 weeks. When categorised by duration and recurrence of symptoms, 67.7% of those with a T1R had an acute single episode while 61.3% of ENL reactions were chronic episodes, lasting more than six months (figure 5). Fifty-four (72%) of the patients required an inpatient stay with a median duration of 35.5 days (25^th^ percentile: 14 days, 75^th^ percentile 105 days).

### Delay in Presenting to Services

Delays were seen between symptom onset and treatment in those attending specialist services (average 12 months, range 0–24 months) and integrated services (average 2.9 months, range 0–12 months), with much of the delay occurring before presentation. Obstacles to early presentation with reaction symptoms included a lack of awareness of the complications of leprosy and regarding health as a low priority when compared to wage earning. Before having a leprosy reaction only four patients reported any previous knowledge of leprosy reactions, two of these as a consequence of an inpatient stay in a specialist leprosy hospital.

*Participant 55, a 32-year-old male from South East Nepal had been aware of skin changes and a tingling sensation in his right hand for two months before he attended Lalgadh Hospital for advice. When asked why he had waited so long to access healthcare he explained that he had been unable to attend sooner as it was essential that he finished planting rice in his fields, that his health was a lower priority than that of feeding his family.*

Eight (12%) of the total patients consulted traditional healers in a range of 1–50 times before seeking care elsewhere. Six received ritualised therapies, one received no treatment and one patient was advised to attend leprosy specialist services.

*Participant 50, a 23-year-old male initially noticed a patch of skin where sensation was abnormal but only sought help when nodules, later diagnosed as ENL, appeared some weeks later. Because of his traditional beliefs he attended the local faith healer who told him he had chicken pox and treated him with a clay paste. Three months later when the nodules continued to be a problem he attended a specialist leprosy hospital on the advice of another leprosy patient from his village.*

### Ongoing Care

Thirty-five patients (47%) received the majority of their MDT from a specialist service, 17 as inpatients and 18 as outpatients. Twenty-three patients (31%) received most of their MDT from a health or sub health post, 13 (17%) from a district or general hospital and two (3%) from other locations. Two patients were diagnosed with leprosy on the day they were interviewed and so had no follow-up information at time of interview.

*Participant 70, a 58-year-old farmer was diagnosed with leprosy and a type 1 reaction earlier this year. He explained that while he was able to collect his MDT at the health post within his village he needed to travel back to the leprosy hospital twice a month to collect steroids, taking a full day each time. When asked why he had to travel to two different locations for follow-up he said he didn't know.*

Thirty-three patients (44%) received the majority of their reaction treatment as outpatients; 25 (76%) at a specialist services, seven (21%) at district or general hospitals and one (3%) at a local health post. Thirty-one patients (41%) were taking MDT and steroids simultaneously, on a predominantly outpatient basis. Twenty-seven patients (87%) experienced no difficulty in receiving reaction medications, while four (13%) reported difficulties related to travel or expense. Of the patients treated on an outpatient basis, 18 patients (58%) were able to access MDT and steroids from the same location but 13 (42%) attended two different locations; one for MDT, a second for steroids. With both MDT and steroids available from specialist services it is not clear why patients opted for this.

## Discussion

Our data shows that in Nepal, patients with leprosy reactions experienced significant delays in accessing reaction treatment. While the average delay was 2.9 months, symptomatic patients went untreated for up to two years, often because they failed to seek help early. Even following presentation to a health service many patients with reactions were not diagnosed or treated correctly. Most of the patients interviewed (59%) who initially presented to an integrated service were not started on treatment at their first consultation. In contrast, the majority of those attending a specialist service (86%) directly were started on steroids at first consultation. These finding suggest a lack of knowledge regarding leprosy reaction amongst patients, communities and healthcare workers which must be tackled to reduce treatment delay and consequent disability.

Leprosy reactions can be severe, recurrent or chronic. 87% of our patients did not have a satisfactory response to a 12 week standardised course of steroids. And 61% of those with ENL had a chronic reaction, a similar rate to that reported in an Indian cohort of 481 leprosy outpatients [9] and inpatient treatment was required by 72%. Those caring for patients with leprosy reactions must be able to monitor treatment response and have a clear plan for those with complex or non-responsive disease.

Steroids were not always readily available in the peripheral setting. Of the 31 outpatients receiving treatment for leprosy and reaction simultaneously, 42% attended a different location to access steroids than that supervising their MDT. The data from an RCT comparing steroids versus placebo suggests that steroids can be used safely in the field setting with an increase in only minor adverse events (RR1.6) [15]. As nerve recovery depends on prompt treatment, concerns regarding non-specialist steroid use must be balanced against risk of disability. A system of partial integration, which would inconvenience patients and threaten treatment compliance, should be avoided.

Patients continue to attend specialist services. Despite peripheral diagnostic and treatment services, 65% of those interviewed were diagnosed by a leprosy specialist service. On developing reaction symptoms 38% presented directly to specialist services including 18 previously unknown patients. Continued presentation to specialist services has been observed in other countries following integration. An evaluation of leprosy services in post-integration Sri Lanka found that while specialist services were diagnosing fewer cases of leprosy, patients were opting to attend institutions classed as secondary or tertiary care despite peripheral availability [16]. Cost may play a role as specialist services are free while some peripheral services charge a consultation fee. Patients may also feel less at risk of stigmatizing behaviour within a specialist service. A qualitative study of post-integration leprosy care in the Indian state of Orissa identified problems with stigmatizing behavior by healthcare staff towards patients with leprosy [17]. Further research to ascertain the factors influencing patient choice in Nepal would provide useful information to help reduce barriers and encourage peripheral service use.

Patients were frequently symptomatic for months prior to presentation. Patient and community education regarding the long-term implications of neglecting symptoms must be improved. As 40% of those interviewed were known leprosy patients, the need to improve education at time of diagnosis and follow-up must be acknowledged. Methods not dependent on literacy such as radio and community-based programmes should be prioritized.

The major limitation of this study was that we interviewed patients already using specialist services. This was a pragmatic decision made so that we could identify and interview enough patients in our time frame. However these patients are likely to be those with more severe reactions. We do not know how many patients are successfully managed in the peripheral setting. However, 65% of our sample self-referred, implying that there was a range of symptom severity. Data on both groups of patients; those managed entirely within the peripheral setting and non-users, would be needed to formally evaluate the integrated service.

This study suggests that the complexity and severity of leprosy reactions may not have been fully recognized during the integration process. With 93% of patients living in a rural location the argument for improving access through integration with peripheral services is compelling. However, peripheral workers must be supported in their expanded role. The expertise of those working in specialist services should be used for consultancy and training to aid peripheral workers to recognize and treat reactions early and to monitor for an adequate treatment response. There must be a clear referral pathway to ensure that those not responding to treatment or with complex reactions are able to access timely specialist input. Equally, to allocate resources effectively and improve access to treatment, the referral pathway back to a peripheral service for those with simple reactions should be optimized. Health policy makers, not only in the field of leprosy, need to be cautious when simplifying healthcare delivery to ensure all healthcare needs are met.
